# Prescription Digital Therapeutics as Living Interventions: Rethinking Evidence, Dosage, and Regulation in Adaptive Medicine

**DOI:** 10.7759/cureus.107184

**Published:** 2026-04-16

**Authors:** Rahul Mittal, Nancy Zhang, Rajnish Mohindroo

**Affiliations:** 1 Health Informatics, Rutgers University, Piscataway, USA; 2 Computer Science and Statistics, Rutgers University, New Brunswick, USA; 3 General Practice, Health and Beyond Partnership, Wolverhampton, GBR

**Keywords:** clinical innovation, healthcare technology, personalized medicine, prescription digital therapeutics, software-based care

## Abstract

Prescription digital therapeutics (PDTs) are transforming healthcare by delivering evidence-based interventions through adaptive software platforms. Unlike traditional therapies, PDTs can evolve after deployment through updates and user-driven modifications, challenging conventional models of evidence generation, dosage definition, and regulatory oversight. This editorial argues that PDTs should be conceptualized as “living interventions,” requiring new frameworks that account for their dynamic nature.

Key challenges include the absence of standardized definitions for digital “dose,” variability in patient engagement, and the difficulty of measuring outcomes in continuously evolving systems. Additionally, the transition of therapeutic care into digital environments raises questions about maintaining therapeutic alliance and patient trust. Existing regulatory pathways, designed for static medical products, are insufficient for technologies that change over time.

We propose the need for interdisciplinary and anticipatory approaches that integrate clinical science, engagement science, regulatory innovation, and implementation strategies. Ethical considerations, including equity, data privacy, and access, must remain central to ensure that PDTs enhance rather than widen healthcare disparities.

As digital therapeutics continue to expand, aligning adaptability with accountability will be critical to their safe, effective, and equitable integration into modern healthcare systems.

## Editorial

Introduction

Prescription digital therapeutics (PDTs) and digital medicines are reshaping modern healthcare delivery and therapeutic design [1,2]. PDTs provide evidence-based and FDA-approved therapeutic interventions to patients through digital platforms, distinguishing them from traditional pharmacologic treatments [2,3]. As emerging technologies transition from controlled research to real-world clinical practice, their pace of evolution increasingly exceeds the speed at which medical research, regulation, and policy can adapt. To address the rapid expansion of digitalized prescriptions, this article argues for the necessity of forward-looking, conceptual, and anticipatory research frameworks [4,5]. Such research should anticipate, respond to, and predict potential problems before they arise, rather than concentrating exclusively on retrospective studies regarding PDTs.

Three interconnected domains highlight why this shift to PDTs requires urgent attention: the problem of defining dosage through adaptive software, the challenge of preserving the essence of therapeutic treatment in digital environments, and the inadequate guardrails of current regulatory pathways for these innovative interventions. Each area must be studied thoroughly before PDTs are fully integrated to prevent recurring problems from emerging from a technology designed to change over time.

PDTs have the modernized capability to receive updates, even after their initial release and deployment [2,5]. Updates, which are unfeasible for standard methods of treatment, take the form of software upgrades, additional content release, or adaptations based on user response [2,5]. A smart treatment that can be corrected and adjusted from the patients' data following distribution has a multitude of prospective benefits [1]. However, there are a few glaring dilemmas, one of the most evident being that scientific publications and institutions favor measurable results: with a continually evolving creation, results that are undeniably variable and must be analyzed in a newfound fashion. Thus, developing clear frameworks can clarify what counts as "evidence" for changing interventions, ensuring accountability and safety [5,6].

Dosage and PDTs

One critical question revolves around the notion of a "digital dose" [2]. In pharmacology, dosage is physically quantifiable, for example, a specific number of pills. In software, defining a dosage is more convoluted. A PDT's effectiveness depends not only on its content, but also on the amount of interaction the patient has with it. The depth of a patient's interaction with a PDT lacks a standardized dosage metric, primarily because of variability in user interaction and the dynamic nature of digital content [2,4]. Exposure may involve time spent on modules, completion rates, behavioral responses, or algorithm-triggered adaptations. Therefore, interdisciplinary collaboration across clinical science and engagement science is necessary to establish standardized definitions of exposure, adherence, and therapeutic response for digital therapeutics [5].

Consider one example of a digital PDT, in this case being reSET and reSET-O, which are PDTs developed by Pear Therapeutics for substance use disorder and opioid use disorder, respectively. They deliver cognitive behavioral therapy (CBT) modules through a mobile app, bundled with standard medication treatment. Patient retention improved, yet secondary measures such as urine drug tests and mood assessments showed no significant differences between groups [7]. This disparity begs the question, what aspect of the digital intervention led to the retention benefit? Was it the number of completed modules, the engagement frequency, the specific content, or something else? A patient who completes every module in short, focused sessions has a different pattern of exposure, or dosage, from one who engages in sporadic sessions, yet both fall under the same branch of treatment.

The dosage problem extends beyond behavioral health as well. In a review of digital therapeutics for irritable bowel syndrome (IBS), researchers looked into FDA-approved PDTs that provide brain-gut behavioral therapies: Mahana, which delivers self-guided cognitive behavioral therapy, and Regulora, which delivers gut-directed hypnotherapy [8]. IBS presents a multidimensional symptom profile, with abdominal pain, quality of life, stool consistency, and more, each representing distinct targets for therapeutic interventions. Clinical trials showed Regulora significantly reduced abdominal pain during treatment, in comparison with a muscle relaxation control, yet showed no significant impact on stool consistency outcomes. Mahana IBS exhibited significant improvements in overall symptom severity, but engagement varied significantly, where completion rates across studies differed extensively [8]. These findings again highlight the question of what constitutes an adequate dose. Without a standardized metric to distinguish these dosage patterns, clinical trials may be unable to observe which dimensions of a PDT truly drive results, and it will be difficult to produce results that can be accurately interpreted, replicated, or compared to other methods of treatment.

Therapy digitalized

The essence and effectiveness of therapy stem from the trust and connection built between a doctor and patient. PDTs offer the convenience of receiving therapy from a screen, but also carry the caveat of relinquishing the genuine human care that is the foundation of therapy. Designing intentional, meaningful elements is essential when transitioning to a digital framework to ensure that patients who use PDTs gain the same benefits as those of traditional therapy [1,2,4].

An increasing amount of literature on digital CBT platforms suggests that personalized feedback, friendly interfaces, and strategic check-ins can influence a patient's satisfaction with being heard and supported [1,2]. Some studies have even found that well-designed digital interventions achieve similar therapeutic alliance scores to traditional in-person therapy, particularly when digital methods are supplemented by brief human contact [1,2]. However, other research indicates that PDTs that lack any human touch see higher dropout rates and lower patient satisfaction [1].

Human factor research, engagement science, and user-experience design must therefore be integrated into PDT development to preserve therapeutic alliance in software-mediated care. The mixed findings above underscore the necessity for the above. Continued research on user interaction and engagement will be critical for maintaining meaningful therapeutic relationships within digital environments [4].

Why rigid rules struggle

The current existing jurisdiction was designed for conventional medical devices and pharmaceuticals that do not update themselves on a continuous basis [5]. Examples of prominent pathways include the FDA's 510(k), De Novo pathways, and the European Union's Medical Device Regulation (MDR), which are key for clearing new medical devices for the market. With PDTs, it is no question that they will also have to undergo the same regulatory process. Because PDTs may undergo iterative software updates, regulators must consider lifecycle-based validation rather than one-time clearance models. Navigating these waters can be tricky, as a plethora of considerations ranging from a PDT's constant, yet necessary, collection of sensitive patient data to the ever-changing and potentially unpredictable model must be taken into account without a basis to fall back upon [4,6]. As innovators, we should support adaptation to the modern medical system, but not at the cost of patient safety and transparency.

Ethics and equity

The growth of PDTs and smart medicine relies on four interconnected areas: clinical, engagement, regulatory, and implementation [2,4,5]. Clinical science explores and examines safety and efficacy. Engagement science investigates usage and sustainability. Regulatory science establishes ethical standards and procedures to ensure patient safety and satisfaction. Implementation determines how the resulting product reaches real patients equitably. With these in mind, further study can integrate these pillars into a unified framework for the continued development of PDTs [4].

Additionally, the ethics and equity considerations surrounding PDTs require thoughtful and thorough deliberation. While the broad-ranging goal is to democratize access to healthcare, there is an underlying risk of achieving the exact opposite: widening disparities for populations with limited access to the digital accessibility that PDTs promise [9,10]. Further questions regarding data privacy, stewardship, and consent arise because PDTs must continually collect personal data and thus must also be addressed [5,6]. Equity-focused design principles should be embedded from the earliest stages of development to ensure access, affordability, and cultural relevance [4,10].

Thinking towards the future of PDTs

The preceding sections of this article share a common theme: in each of the discussed domains, the current schema of retrospective evaluation is insufficient for a new technology that changes after deployment. Yes, PDTs bring a new way of thinking and expectation for the future of therapy and prescriptions, and it is very appealing that medical-grade treatment can now be accessed from the comforts of one's own home and device [1,2].

However, we must tread forward with caution, as always with newfound trails. The field of medicine is supported by research and evidence, but the human experience and meaning of healthcare rely on the expectation that ethics, equity, and trust remain indispensable [4,5]. Digital therapeutics and smart medicines are intended to enhance, not replace, the human dimensions of care. Future progress will require anticipatory frameworks that balance adaptability with accountability, ensuring that technological advancement strengthens rather than destabilizes modern healthcare systems.
